# Serine/threonine kinase 3 promotes oxidative stress and mitochondrial damage in septic cardiomyopathy through inducing Kelch-like ECH-associated protein 1 phosphorylation and nuclear factor erythroid 2-related factor 2 degradation

**DOI:** 10.7150/ijbs.80800

**Published:** 2023-02-21

**Authors:** Hang Zhu, Zhe Dai, Xiaoman Liu, Hao Zhou, Yijin Wang

**Affiliations:** 1 School of Medicine, Southern University of Science and Technology, Shenzhen, Guangdong, China.; 2 Senior Department of Cardiology, The Sixth Medical Center of People's Liberation Army General Hospital, Beijing, China.

**Keywords:** STK3, KEAP1, Nrf2, mitochondria, septic cardiomyopathy.

## Abstract

Serine/threonine kinases (STK3) is a core component of the Hippo pathway and modulates oxidative stress and inflammatory responses in cardiovascular diseases. However, its potential role in septic cardiomyopathy remains undefined. STK3-mediated phosphorylation of Kelch-like ECH-associated protein 1 (KEAP1) was shown to suppress antioxidant gene transcription controlled by nuclear factor erythroid 2-related factor 2 (Nrf2) in macrophages. To explore whether STK3 induces KEAP1-mediated suppression of Nrf2 in septic cardiomyopathy, wild-type and global STK3 knockout (STK3*^-/-^*) mice were treated with LPS. LPS treatment upregulated cardiac STK3 expression. STK3 deletion attenuated myocardial inflammation and cardiomyocyte death, and improved myocardial structure and function. In LPS-challenged HL-1 cardiomyocytes, shRNA-mediated STK3 knockdown normalized mitochondrial membrane potential and ATP production, attenuated apoptosis, and rescued antioxidant gene expression by preventing Nrf2 downregulation. Co-IP, docking analysis, western blotting, and immunofluorescence assays further showed that STK3 binds and phosphorylates KEAP1, promoting Nrf2 downregulation. Accordingly, transfection of phosphodefective KEAP1 mutant protein in cardiomyocyte restored Nrf2 expression and mitochondrial performance upon LPS, while expression of a phosphomimetic KEAP1 mutant abolished the mitochondria-protective and pro-survival effects of STK3 deletion. These findings suggest that STK3 upregulation contributes to septic cardiomyopathy by phosphorylating KEAP1 to promote Nrf2 degradation and suppression of the antioxidant response.

## Introduction

An excessive and unresolved immune response underlies multiple organ dysfunction and high mortality in patients with septic shock. Septic cardiomyopathy is a severe complication of sepsis and septic shock, associated with a mortality rate of ~50% 1. Despite advances in diagnostic methods, the pathophysiological mechanisms of septic cardiomyopathy remain incompletely understood 2. Cardiomyocytes contain a high number of mitochondria, which are vital to sustain cardiac contraction/relaxation cycles. Numerous studies concur in that mitochondrial dysfunction in cardiomyocytes is a pivotal event during septic cardiomyopathy 3, 4. Decreased mitophagy, increased mitochondrial fission, excessive production of reactive oxygen species (ROS), and impaired mitochondrial metabolism have been observed in a series of cellular and animal studies addressing the mechanisms underlying sepsis-related organ failure 5-7. However, the upstream mediators of mitochondrial damage in septic cardiomyopathy remain incompletely characterized 8.

Serine/threonine kinase 3 (STK3), also known as mammalian sterile 20-like kinase 1 (Mst1), is a core component of the Hippo pathway affecting cellular apoptosis and oxidative stress. Several substrates of STK3 have been reported, including glucose transporter 1 (GULT1) 9, mitogen-activated protein kinase (MAPK) 10, NLR family pyrin domain containing 3 (NLRP3) inflammasome 11, leukotriene B4 (LTB4) 12, and salvador homolog 1 (SAV1) 13. Previous findings have uncovered the pathological action offered by STK3 in various cardiovascular diseases and conditions, such as diabetic cardiomyopathy 14, oxidative stress-Induced cardiomyocyte differentiation and death 15, adrenergic cardiomyopathy 16, and stress-induced myocardial hypertrophy 17. However, the potential involvement of STK3 in septic cardiomyopathy has not been determined.

Although STK3 are mainly expressed in the cytoplasm, ample evidence attests to their influence on mitochondrial homeostasis. After myocardial ischemia-reperfusion (I/R) injury, the expression of STK3 is significantly elevated and correlates with decreased mitochondrial function, as evidenced by increased mitochondrial fission and prolonged opening of the mPTP 15. Besides, a regulatory role for STK3 on mitochondrial metabolism 18 and mitophagy 19 has been described in different types of cells. For instance, STK3 deletion in macrophages was shown to impair post-infarction cardiac repair 12, and reduce their bactericidal activity 11. In these cells, STK3 serve as a ROS sensor and STK3 deficiency results in increased oxidative injury, senescence, and death. The underlying mechanism involves changes in the phosphorylation status of Kelch-like ECH-associated protein 1 (KEAP1) and enhanced degradation of Nrf2, a master regulator of the cellular antioxidant defense 20. In turn, assays in cultured HEK-293 indicated that hydrogen peroxide-mediated STK3/4 activation promotes a positive feedback loop that sustains an oxidizing cellular state by phosphorylating and inactivating peroxiredoxin-2 (Prdx2), an important regulator which suppresses the oxidative stress in cardiomyocyte through promoting the conversion of hydrogen peroxide to water 21. Based on the above evidence, indicative of STK3-mediated regulation of mitochondrial homeostasis and the presence of a STK3/KEAP1/Nrf2 signaling axis in macrophages governing Nrf2-dependent antioxidant gene expression, we investigated the potential impact and mechanism-of-action of STK3 in animals and cells models of septic cardiomyopathy.

## Materials and methods

### Animals and echocardiography

All animal experiments were approved by the Animal Care Committee of the Southern University of Science and Technology and the Sixth Medical Center of People's Liberation Army General Hospital. Global STK3 knockout (STK3*^-/-^*) mice were purchased from Jackson Laboratory (Strain #017635). To induce septic cardiomyopathy, STK3*^-/-^* and wild-type (WT) mice were injected with LPS (10 mg/Kg, 12 hrs) 22. Myocardial performance was determined by echocardiography 23 using a Vevo 3100 (FUJIFILM Visual sonics, Toronto, Canada).

### Immunohistochemistry, H&E staining and electron microscopy

Heart tissues were fixed and processed for histopathological examination. De-paraffinized and rehydrated samples were heated and 3% H_2_O_2_ was used. Then, samples were incubated with NF-κB antibody (1:500, #ab32536, Abcam). After treated with PBS, samples were treated with HRP-conjugated rabbit anti-goat IgG for 20 min, washed, and exposed to 3,3ʹ-diaminobenzidine tetrahydrochloride (DAB). H&E staining was performed on 10% formalin-fixed heart sections following standard procedures. Electron microscopy was observed under a JEM 1016CX electron microscope (JEOL, Tokyo, Japan).

### Immunofluorescence

Heart tissue sample was cut with a Leica CM3050S cryostat, air-dried, and fixed in 10% formalin. After washing with PBST, samples were treated with antibodies (Gr1, 1:500, #14-5931-82, Invitrogen, Carlsbad, CA) 24. The samples were then washed with PBS and then treated with Alexa Fluor 488-conjugated Goat anti-Rabbit IgG (H+L) (Invitrogen) for 1 h. Images were captured by a Motorized Zeiss Axio Observer Z1 inverted microscope at 40x magnification. The images were organized by Zen 3.2 software and processed by gramma adjustment. Gr1^+^ staining was quantified using Image J software. TUNEL staining was conducted through a TUNEL Assay Kit (#C10617, Invitrogen) 25.

### ELISA

Concentrations of SOD (Mouse Superoxide Dismutase ELISA Kit, #MBS034842, MyBioSource, Inc.), GSH (Mouse Superoxide Dismutase ELISA Kit, #MBS034842, MyBioSource, Inc.), GPX (Mouse glutathione peroxidase ELISA Kit, #CSB-E13068m, Cusabio, Inc.), PRX1 (Mouse Peroxiredoxin-1 (Prdx1) ELISA Kit, #MOEB2015, AssayGenie, Inc.) and Caspase-3 activity (Mouse CASP3 ELISA Kit, #E4591, BioVision, Inc.) in cell homogenates were determined by ELISA according to the manufacturers' protocols 26.

### Cell culture and transfection procedures

To establish stable STK3 knockdown, HL-1 cells were infected with short hairpin RNA (shRNA) targeting STK3 (shSTK3; obtained from AMS Biotechnology (Europe) Ltd, #SH805830) 27. To establish stable Nrf2 knockdown, HL-1 cells were infected with shRNA targeting Nrf2 (shNrf2; obtained from Santa Cruz Biotechnology, Inc., #sc-37049-V). Non-silencing shRNAs were used as control. Stable STK3/Nrf2 knockdown cells were selected with puromycin (3 μg/mL) over two weeks. To mimic septic cardiomyopathy, HL-1 cells were exposed to LPS (10 μg/ml) for 6 hrs min prior to experimental determinations 28.

### Cell viability

STK3 knockdown (shSTK3) HL-1 cells and control (shNC) cells were seeded at 4 x 10^4^ cells/well. After LPS stress, cell survival and function were determined using a Cell Counting Kit-8 (Beyotime Biotechnology, C0042) 29.

### Immunoprecipitation and docking analysis

Immunoprecipitation assays were performed as described before 30. Immunoprecipitation reaction was made overnight with 1mg of cell extract, dissolved in 400 μL of lysis buffer (composition described above) 2.5 mM DTT, 30 μL of glutathione agarose beads and 5 μg of antibody. Docking analysis was performed to obtain a set of possible conformations and orientations for the ligand at the binding site using AutoDock Vina software (https://vina.scripps.edu/). Protein information was converted into a PDBQT file.

### Mitochondrial membrane potential, ROS, and calcium detection

Mitochondrial potential was observed using a JC-1 Assay Kit (Cat. No.: HY-K0601, edChemExpress) 31. ROS in mitochondria or cytoplasm were reacted by a Mitochondrial Superoxide Indicators (#M36008, Invitrogen) or a H_2_DCFDA probe (Cat. No.: C263, ABP Biosciences), respectively. ATP concentration in cardiomyocyte was evaluated through bioluminescence using an ATP Kit (#MAK190, Sigma-Aldrich). Mitochondrial calcium was detected through Rhod-2 (#R1244, Invitrogen) 32.

### Quantitative PCR

Total RNA was isolated from mouse ventricles using TRIzol Reagent (Invitrogen). RNA molecules were reverse transcribed using Superscript III Reverse Transcriptase kits (Invitrogen) 33. The primers used for qPCR were: *IL-6* (Forward, 5'-CAACGATGATGCACTTGCAGA-3'; Reverse, 5'-GTGACTCCAGCTTATCTCTTGGT-3'), *TNFα* (Forward, 5'-TGATCGGTCCCCAAAGGGAT-3'; Reverse, 5'-TGTCTTTGAGATCCATGCCGT-3'), *MMP9* (Forward: 5'-CATTCGCGTGGATAAGGA-3', Reverse, 5'-ACAAGAAAGGACGGTGGG-3'), *MCP1* (Forward, 5'-TGATCCCAATGAGTAGGCTGGAG-3'; Reverse, 5'-TGTCTGGACCCATTCCTTCTTG-3').

### Immunoblot analysis

Total protein extracts were prepared by homogenization of mouse ventricles in Urea lysis buffer. Samples were separated on 4-12% SDS-PAGE gels (Life Technologies) and incubated with primary antibodies, including STK3 (1:1500, #ab52641, Abcam), KEAP1 (1:1500, #ab227828, Abcam), Bax (1:2000, #ab32503, Abcam), Bcl2 (1:1500, #ab182858, Abcam), and Nrf2 (1:1500, #ab92946, Abcam). Immunoreactive protein bands were visualized using ECL reagent. The representative image for each group was selected to show the average or median level of the group based upon the mean value 34.

### Statistical analysis

Data are presented as mean ± SEM. P < 0.05 was statistically significant. One-way ANOVA adjusted by Tukey's post hoc test and Kruskal-Wallis adjusted by FDR's post hoc test were used for statistical tests of pairwise multiple comparisons. Statistical analysis was performed using R package v3.6 and GraphPad Prism v8.0.2.

## Results

### Cardiac STK3 expression is increased in LPS-mediated septic cardiomyopathy and correlates with decreased heart function

**Figure 1:**
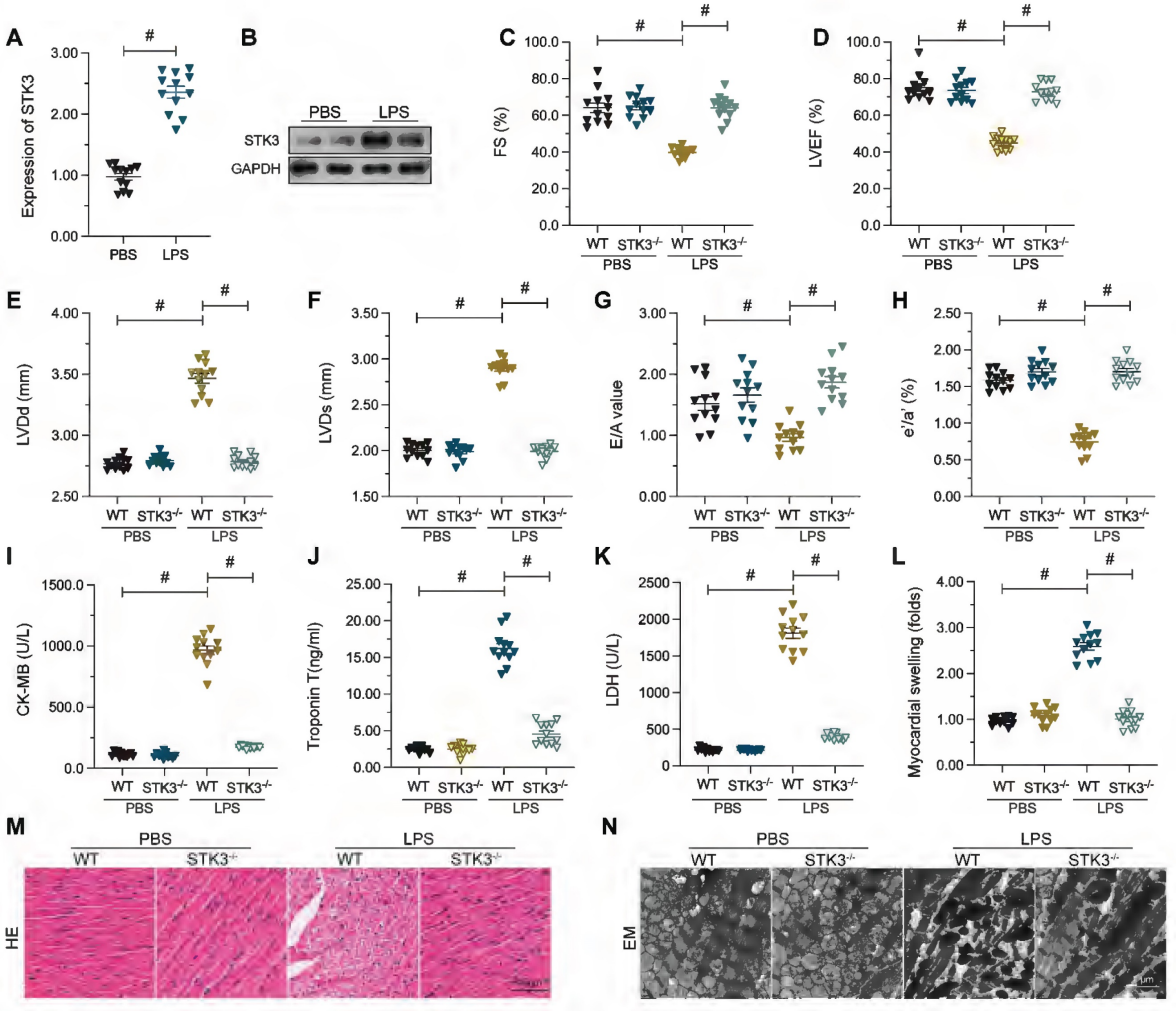
** Increased cardiac STK3 expression correlates with decreased heart function in mice with LPS-induced septic cardiomyopathy.** Wild-type (WT) and STK3^ -/-^ mice were injected with LPS (10 mg/Kg) to induce septic cardiomyopathy *in vivo.* Echocardiography, biochemical, and histological analyses were conducted 12 h later. **(A, B)** Western blot analysis of STK3 expression in cardiac tissues. **(C-H)** Echocardiography results. LVEF, left ventricular ejection fraction; FS, left ventricular fraction shortening; LVDs, left ventricular systolic dimension; E/A, ratio of early to late transmitral flow velocities; e'/a', ratio of diastolic mitral annulus velocities; LVDd, left ventricular diastolic dimension. **(I-K)** ELISA-based analysis of serum TnT, CK-MB, and LDH levels.** (L, M)** Histopathological analysis via H&E staining. **(N)** Electron microscopy (EM) analysis of myocardial ultrastructure. #p<0.05.

To assess potential changes in cardiac STK3 expression during septic cardiomyopathy, western blots were conducted in mouse heart tissues 48 h after a single injection of LPS. Compared to the control group (mice injected with PBS), significantly elevated expression of STK3 was observed in the hearts of LPS-treated wild-type (WT) mice (Figure 1A and 1B). To evaluate the potential impact of STK3 expression on cardiac function and structure in septic cardiomyopathy, global STK3 double knockout (STK3*^-/-^*) mice were injected with LPS. After exposure to LPS, heart systolic function was impaired in WT mice. This was evidenced by decreased left ventricular ejection fraction (LVEF), attenuated rate of left ventricular fraction shortening (FS), and increased left ventricular systolic dimension (LVDs) (Figure 1C-1H). Besides, cardiac relaxation capacities, assessed by estimating ratio of early to late transmitral flow velocities (E/A), ratio of diastolic mitral annulus velocities (e'/a'), and left ventricular diastolic dimension (LVDd), were also blunted in LPS-treated WT mice (Figure 1C-H). In contrast, these alterations were not prominent in STK3*^-/-^* mice (Figure 1C-1H).

ELISA was used next to analyze serum alterations of the myocardial damage markers TnT, CK-MB, and LDH. As shown in Figure 1I-K, LPS injection significantly elevated TnT, CK-MB, and LDH concentration in WT but not in STK3*^-/-^* mice. Consistently, H&E staining showed that LPS promoted myocardial swelling in WT mice, while these alterations were relieved in STK3*^-/-^* mice (Figure 1L-M). Similarly, electron microscopy (EM) displayed distorted myocardial fibers and swollen mitochondria in WT mouse hearts (Figure 1N). These structural abnormalities were not evident, however, in STK3*^-/-^* mice (Figure 1L-N). These results suggest that LPS-induced STK3 expression exacerbates sepsis-related myocardial damage.

### STK3 promote myocardial inflammation and cardiomyocyte death during septic cardiomyopathy

**Figure 2:**
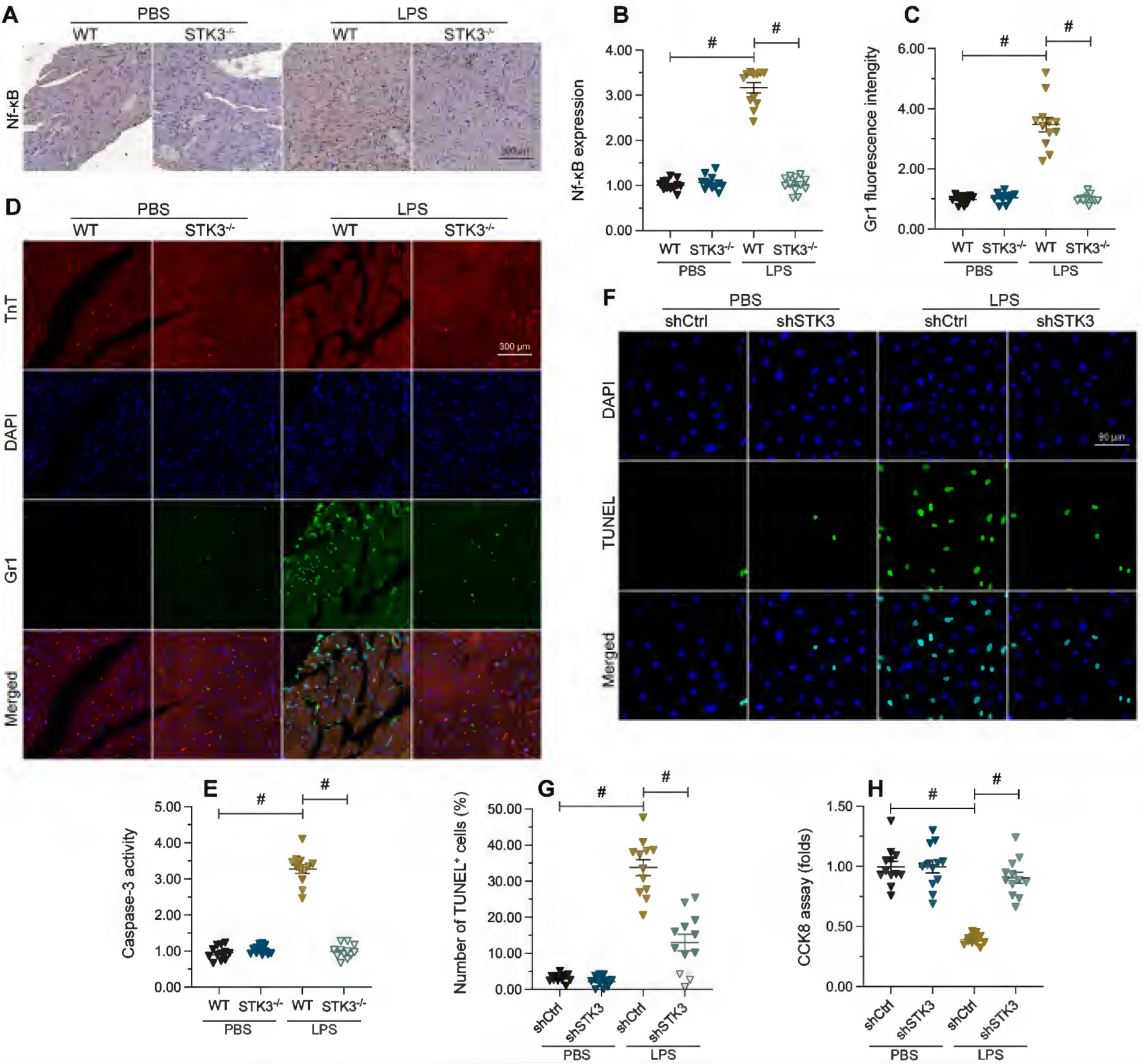
** LPS-mediated STK3 expression promotes myocardial inflammation and cardiomyocyte death.** Wild-type (WT) and STK3^ -/-^ mice were injected with LPS (10 mg/Kg) to induce septic cardiomyopathy *in vivo.* **(A, B)** Immunohistochemical staining of NF-κB in myocardium. **(C, D)** Immunofluorescence staining of Gr-1, a surface marker of neutrophils, in heart tissue. **(E)** ELISA-based detection of caspase-3 activity in heart tissues. **(F, G)** Analysis of apoptosis by TUNEL staining in HL-1 cells treated with LPS (10 μg/ml) to mimic septic cardiomyopathy *in vitro*. Non-silencing (control) and STK3-targeted short hairpin RNA (shRNA) was alternatively transfected into HL-1 cells prior to LPS exposure. **(H)** Analysis of HL-1 cell viability (CCK-8 assay). *p<0.05.

An abnormal myocardial inflammatory response is a known trigger of septic cardiomyopathy. NF-κB is a central mediator of pro-inflammatory gene expression 35. Immunohistochemical staining showed that LPS treatment promoted cardiac expression of NF-κB in WT mice, but not in STK3*^-/-^* mice (Figure 2A and 2B). Furthermore, immunofluorescence staining illustrated that after LPS treatment, the expression of Gr-1, a surface marker of neutrophils, was elevated in heart tissues from WT but not from STK3^-/-^ mice (Figure 2C and 2D).

Cell death of cardiomyocyte is reported to be a key factor aggravating and augmenting sepsis-related myocardial dysfunction 36. ELISA results showed that in response to LPS, the activity of caspase-3, a major cell death-executing enzyme, was elevated in WT heart samples, while STK3 deficiency abrogated this effect (Figure 2E). To validate this finding, we performed STK3 knockdown in HL-1 cardiomyocytes via transfection of STK3-targeted shRNA (shSTK3) and evaluated LPS-induced apoptosis by TUNEL assay (Figure 2F-G). Following exposure to LPS, an apoptosis rate of ~34% was recorded in control cells, while the apoptotic index was reduced to ~16% in cells transfected with shSTK3 (Figure 2F and 2G). Consistent with these findings, CCK8 assay data illuminated that compared to the control group, shSTK3 transfection improved the cell function and survival of LPS-treated HL-1 cells (Figure 2H). These data indicate that STK3 ablation significantly reduces sepsis-related myocardial inflammation and cardiomyocyte death.

### STK3 induces mitochondrial dysfunction in LPS-treated cardiomyocytes

**Figure 3:**
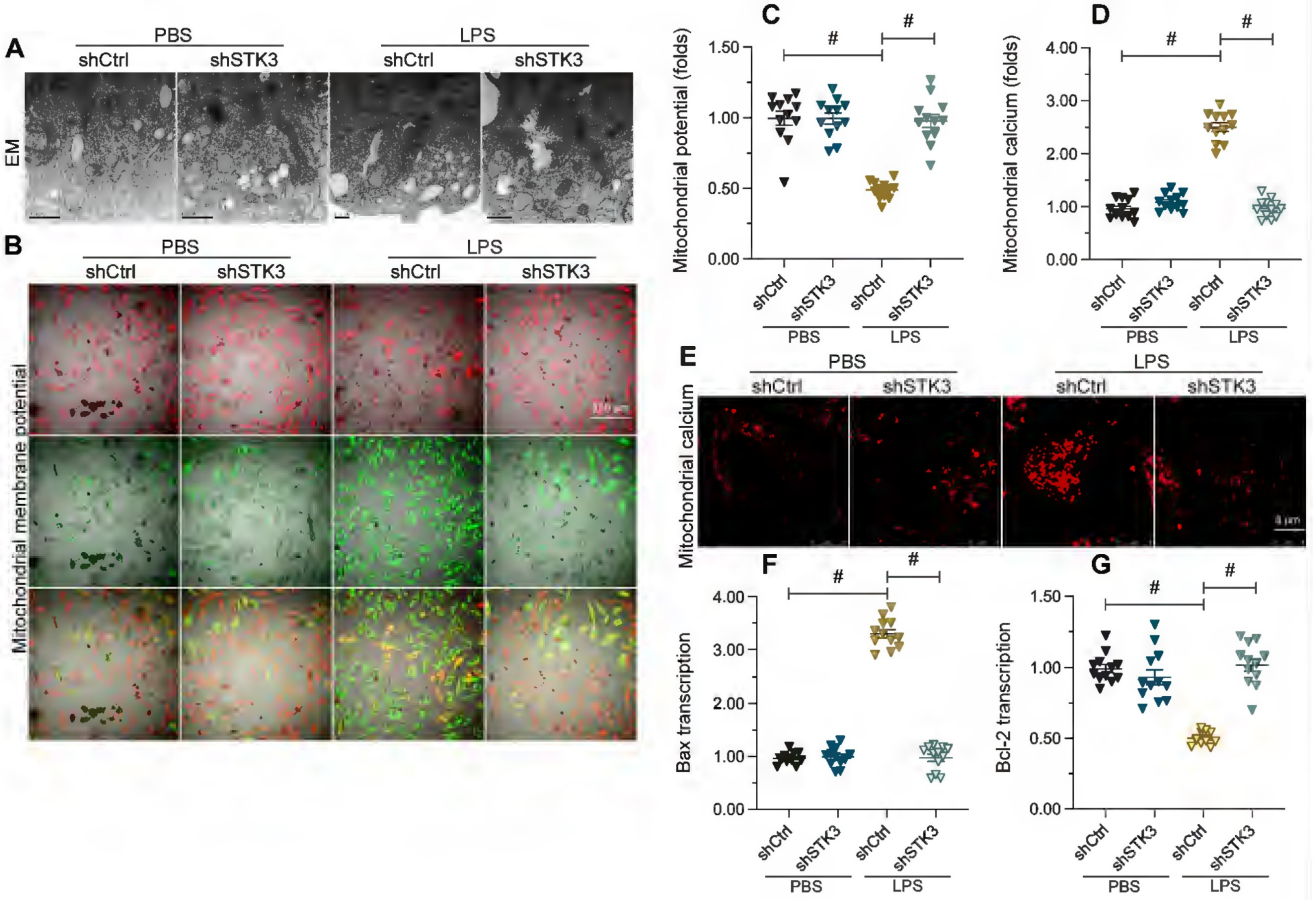
** STK3 deletion prevents LPS-mediated mitochondrial dysfunction in cardiomyocytes.** Wild-type (WT) and STK3^ -/-^ mice were injected with LPS (10 mg/Kg) to induce septic cardiomyopathy *in vivo.* HL-1 cells were treated with LPS (10 μg/ml) to mimic septic cardiomyopathy *in vitro*. Non-silencing (control) and STK3-targeted short hairpin RNA (shRNA) was alternatively transfected into HL-1 cells prior to LPS exposure.** (A)** Electron microscopy (EM) analysis of alterations in mitochondrial ultrastructure in HL-1 cells.** (B, C)** Analysis of mitochondrial membrane potential in HL-1 cells loaded with JC-1. **(D, E)** Mitochondrial calcium detection in HL-1 cells loaded with Rhod-2. **(F, G)** qPCR analysis of Bax and Bcl-2 transcription in cultured HL-1 cells. #p<0.05.

Mitochondrial dysfunction is a prevalent molecular determinant of the pathological alterations underlying septic cardiomyopathy. Therefore, we performed both ultrastructural analysis and molecular assays to evaluate whether STK3 expression contributes to sepsis-related myocardial injury through disruption of mitochondrial homeostasis. Alterations in mitochondrial morphology were first assessed in HL-1 cells through electron microscopy. In Figure 3A, LPS caused mitochondrial swelling with fractured cristae, as well as generation of numerous round and small mitochondria. However, these structural abnormalities were largely absent in cells transfected with shSTK3 (Figure 3A). In addition to morphological alterations, analysis of JC-1 fluorescence indicated that mitochondrial membrane potential was also reduced after exposure to LPS in control but not in STK3-deficient HL-1 cells (Figure 3B and 3C). In line with these observations, immunofluorescence staining further showed that mitochondrial calcium was significantly elevated by LPS treatment in normal cardiomyocyte but remained at physiological levels after shSTK3 transfection (Figure 3D and 3E). Lastly, and confirming the protective effect of STK3 deletion on LPS-mediated apoptosis of cardiomyocytes, qPCR assays showed that shSTK3 transfection abrogated the LPS-caused Bax accumulation and Bcl-2 downregulation observed in control cells (Figure 3F-3G). These data suggest the functional importance of STK3 in sepsis-induced mitochondrial dysfunction in cardiomyocytes.

### STK3 silencing attenuates LPS-mediated oxidative stress in cardiomyocytes by preventing Nrf2 downregulation

**Figure 4:**
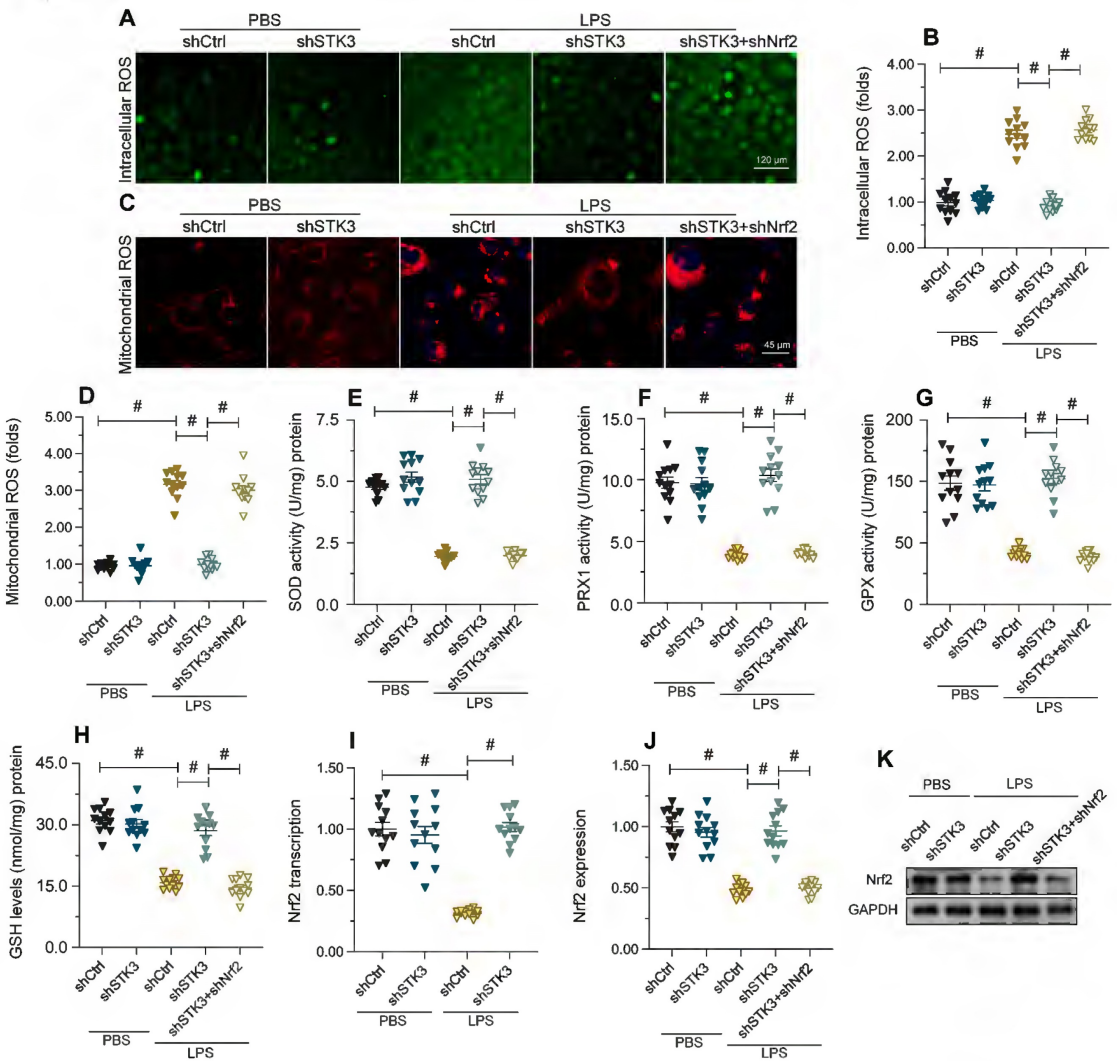
** STK3 deletion attenuates LPS-mediated oxidative stress in cardiomyocytes by restoring Nrf2 transcription.** HL-1 cells were treated with LPS (10 μg/ml) to mimic septic cardiomyopathy *in vitro*. Non-silencing (control) and STK3-targeted short hairpin RNA (shRNA) was alternatively transfected into HL-1 cells prior to LPS exposure. Besides, non-silencing control shRNA and Nrf2-targeted shRNA (shNrf2) was alternatively transfected into HL-1 cells prior to LPS exposure. **(A, B)** Detection of cellular ROS production in HL-1 cells loaded with H_2_DCFDA. **(C, D)** Representative images of HL-1 cells loaded with the mitochondrial ROS indicator MitoSox Red. **(E-H)** ELISA-based analysis of SOD, GSH, GPX, and PRX1 levels in cell homogenates. **(I)** Analysis of Nrf2 transcriptional expression by qPCR. **(J, K)** Western blot analysis of Nrf2 expression. #p<0.05.

Since oxidative stress is a main trigger of mitochondrial dysfunction 37, we next explored whether STK3 expression influences LPS-induced oxidative stress. First, we examined cellular ROS levels in HL-1 cells loaded with H_2_DCFDA. As shown in Figure 4A and 4B, LPS treatment increased ROS production, while this effect was ameliorated by shSTK3 transfection. Likewise, mitochondrial ROS production (mtROS), assessed with the MitoSox probe, was also elevated by LPS and attenuated by shSTK3 knockdown (Figure 4C and 4D). These results indicated that STK3 deficiency attenuated LPS-related redox imbalance in cultured cardiomyocytes. In agreement with these findings, ELISA results showed that during LPS challenge the content of anti-oxidative enzymes, i.e. SOD, GSH, GPX, and PRX1, was significantly downregulated in control HL-1 cells, while shSTK3 transfection reversed this effect (Figure 4E-4H). Considering that the transcriptional expression of antioxidant enzymes is primarily regulated by Nrf2, we therefore investigated whether, following LPS exposure, STK3 impairs cellular antioxidant capacity by influencing the expression of Nrf2. qPCR and Western blot analysis showed that upon LPS exposure, Nrf2 transcription (Figure 4I) and expression (Figure 4J-K) were obviously downregulated in control but not in shSTK3-transfected HL-1 cells (Figure 4I and 4J). To confirm that normalized Nrf2 expression contributes to cellular antioxidant protection in LPS-exposed, STK3-deficient cells, we carried out experiments in cells co-transfected with shSTK3 and Nrf2-targeted shRNA (shNrf2) (Figure 4J-K). Loss of Nrf2 promoted both global ROS (Figure 4A-4B) and mitochondrial ROS production (Figure 4C-4D) and also abrogated also the rescuing effect of STK3 silencing on SOD, GSH, GPX, and PRX1 expression (Figure 4E-4H) in LPS-treated HL-1 cells. These results showed that STK3 deletion effectively restores antioxidant capacity in LPS-challenged cardiomyocytes by preventing Nrf2 downregulation.

### STK3 suppresses Nrf2 activity by phosphorylating KEAP1

**Figure 5:**
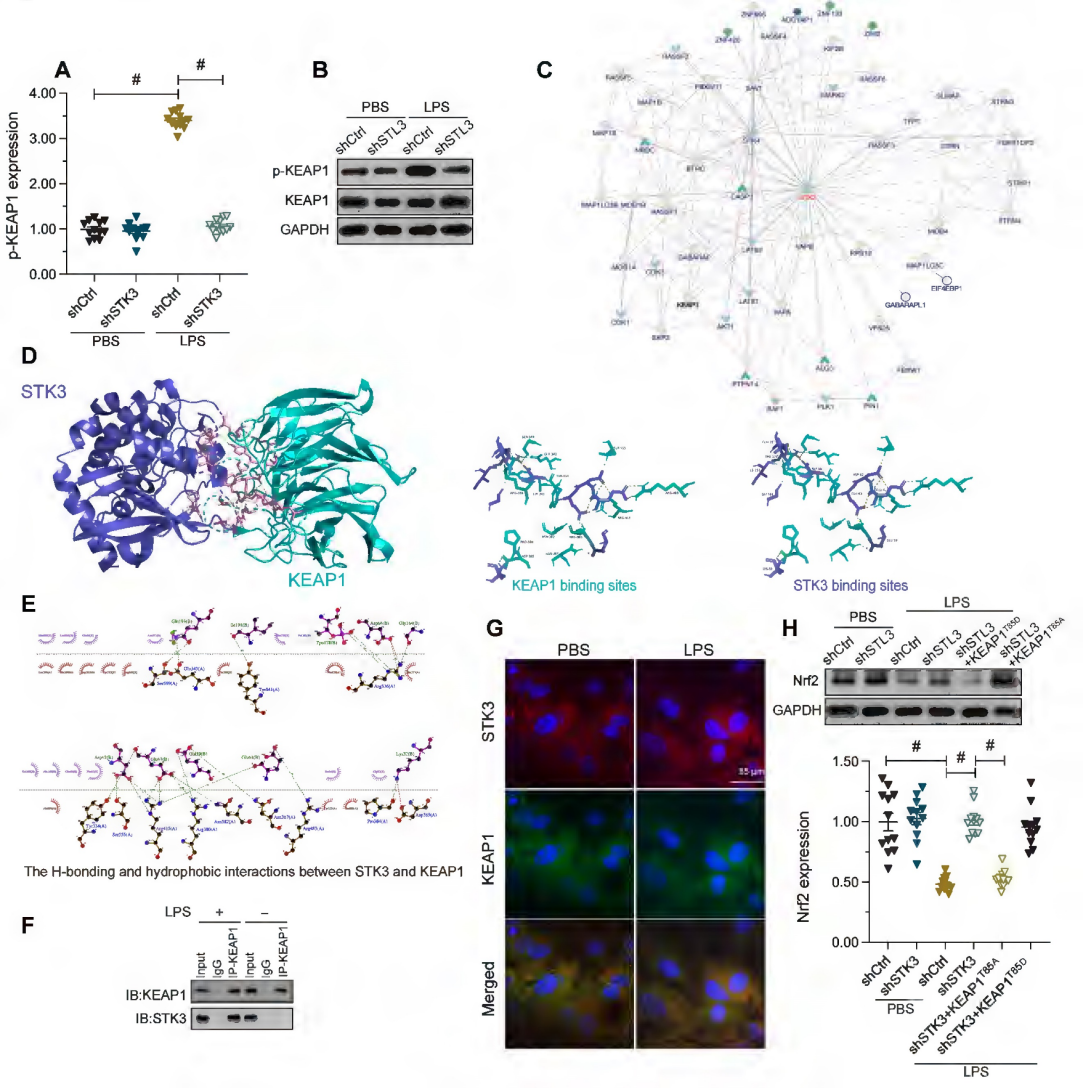
** STK3 interacts with and phosphorylates KEAP1.** HL-1 cells were treated with LPS (10 μg/ml) to mimic septic cardiomyopathy *in vitro*. Non-silencing (control) and STK3-targeted short hairpin RNA (shRNA) was alternatively transfected into HL-1 cells prior to LPS exposure.** (A, B)** Western blot analysis of KEAP1 phosphorylation in LPS-stimulated HL-1 cardiomyocytes. **(C)** Predicted interaction between STK3 and KEAP1, based on analysis in the inBio Discover platform. **(D, E)** Computational docking analysis of STK3-KEAP1 binding. H-bonding and hydrophobic interactions between STK3 and the active region of KEAP1 are shown. **(F)** Co-IP analysis of the interaction between STK3 and KEAP1. **(G)** Representative images of double-immunofluorescence staining indicating interaction between STK3 and KEAP1. **(H)** Western blot analysis of Nrf2 expression in HL-1 cells expressing phosphodefective (KEAP1^T85A^) or phosphomimetic (KEAP1^T85D^) KEAP1 mutant proteins. #p<0.05.

Nrf2 activity has been identified as a downstream effector of Kelch-like ECH-associated protein 1 (KEAP1), an actin-bound cytosolic protein that promotes Nrf2 degradation 38. It has been reported that mutation of T85 in KEAP1 reduces Nrf2 degradation and therefore augments Nrf2-dependent anti-oxidative capacities 39, which suggests that KEAP1 phosphorylation may enhance Nrf2 degradation. Since STK3 has protein kinase activity, we therefore questioned whether STK3 promoted Nrf2 degradation through phosphorylation of KEAP1. Western blots showed that LPS induced KEAP1 phosphorylation in HL-1 cells, and this alteration was reversed by shSTK3 transfection (Figure 5A and 5B). We thus hypothesized that STK3 directly binds and phosphorylates KEAP1. Supporting this hypothesis, the interaction between STK3 and KEAP1 was first predicted by inBio Discover analysis (https://inbio-discover.com) (Figure 5C) and subsequently verified through molecular docking analysis (Figure 5D-5E), which indicated the existence of both H-bonding and hydrophobic interactions between STK3 and the active region of KEAP1, with a minimum binding energy of -12.6 kcal·mol^-1^. Furthermore, co-IP assay results demonstrated that LPS exposure promoted the interaction between STK3 and KEAP1 (Figure 5F), a finding verified also through immunofluorescence (Figure 5G).

To confirm whether STK3 suppresses Nrf2 expression via KEAP1 phosphorylation, phosphodefective or phosphomimetic KEAP mutant proteins (KEAP1^T85A^ and KEAP1^T85D^, respectively) were alternatively transfected into shSTK3-expressing HL-1 cells. Following LPS exposure, western blot assays showed that KEAP1^T85A^ transfection prevented Nrf2 downregulation, while this effect was abrogated by KEAP1^T85D^ transfection (Figure 5H). These results indicated that STK3 negatively regulates Nrf2 expression through KEAP1 phosphorylation.

### Constitutive KEAP1 phosphorylation abolishes STK3 deletion-mediated protection against LPS-induced mitochondrial dysfunction and cardiomyocyte death

**Figure 6:**
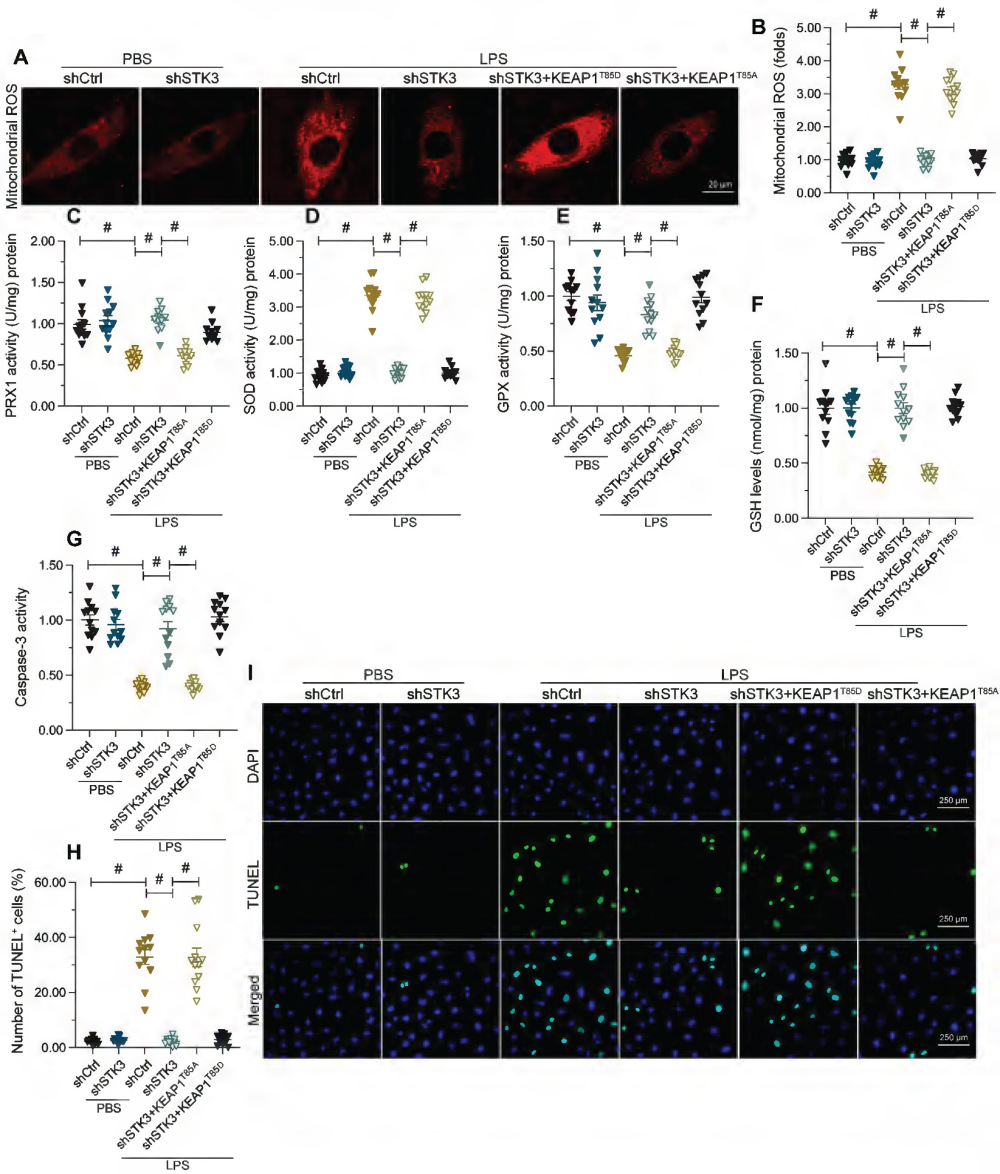
** Constitutive KEAP1 phosphorylation abolishes STK3 deletion-mediated protection against LPS-induced mitochondrial dysfunction and cardiomyocyte death.** HL-1 cells were treated with LPS (10 μg/ml) to mimic septic cardiomyopathy *in vitro*. Non-silencing (control) and STK3-targeted short hairpin RNA (shRNA) was alternatively transfected into HL-1 cells prior to LPS exposure. Phosphodefective (KEAP1^T85A^) or phosphomimetic (KEAP1^T85D^) KEAP1 mutants were transfected into cells in the presence of shSTK3. **(A, B)** Representative images of mitochondrial ROS detection in HL-1 cardiomyocytes. **(C-F)** ELISA-based analysis of SOD, GSH, GPX, and PRX1 in HL-1 cell homogenates. **(G)** ELISA-based analysis of caspase-3 activity. **(H, I)** Assessment of apoptosis in HL-1 cardiomyocytes by TUNEL staining. #p<0.05.

Lastly, we asked whether defective KEAP1 phosphorylation mediates the protective effect of STK3 deletion against mitochondrial dysfunction and cardiomyocyte apoptosis induced by LPS. To validate this possibility, mitochondrial function was first examined in HL-1 cells co-transfected with shSTK3 and KEAP1^T85D^. Results showed KEAP1^T85D^ expression nullified the normalizing effects of shSTK3 on mitochondrial ROS production (Figure 6A and B) and antioxidant enzyme expression (Figure 6C-6F) in LPS-challenged cells. Suggesting also a fundamental role for STK3-mediated KEAP1 phosphorylation in LPS-induced cardiomyocyte death, expression of the phosphomimetic KEAP1^T85D^ protein restored caspase-3 activity (Figure 6G) and promoted apoptosis, as detected by TUNEL staining (Figure 6H and 6I), in cultured HL-1 cells.

## Discussion

The present study addressed the molecular mechanisms underlying septic cardiomyopathy by analyzing the impact of STK3 expression on myocardial function and structure in LPS-treated mice and on mitochondrial dynamics and function in LPS-stimulated, cultured cardiomyocytes. Our study has three main findings. First, STK3 is overexpressed in the heart of mice with LPS-induced septic cardiomyopathy, and ablation of STK3 relieves heart dysfunction, myocardial inflammation, and cardiomyocyte death associated with this condition. Second, STK3-silencing alleviates mitochondrial dysfunction by reversing LPS-mediated Nrf2 downregulation, which prevents in turn oxidative stress injury. Third, STK3 directly binds and phosphorylates KEAP1, which promotes Nrf2 degradation and thus decreases the antioxidant capacity of cardiomyocytes. To our knowledge, this is the first study to identify the STK3-KEAP1-Nrf2-mitochondria pathway as a novel regulator of septic cardiomyopathy. This finding may lead to newer therapeutic approaches targeting STK3 upregulation, KEAP1 phosphorylation, and/or Nrf2 degradation to prevent or treat septic cardiomyopathy.

Mitochondrial dysfunction and redox imbalance stress are crucial molecular alterations in the pathological alterations of sepsis-related myocardial damage. Accordingly, antioxidant therapies and pharmacological protection of mitochondrial homeostasis have shown to relieve myocardial damage during sepsis 40, 41. In endotoxin-induced cardiomyopathy, administration of Vitamin C, a potent antioxidant, is able to reduce the expression of proinflammatory factors, maintain mitochondrial function, and preserve heart performance 42. Supplementation of β-hydroxybutyrate before LPS treatment effectively inhibited histone deacetylase (HDAC) and thus activated the antioxidant RoxO3a/MT2 pathway, which improved both mitochondrial respiration and cardiac output 43. Irisin, an exercise-induced myokine, has been reported to reduce oxidative stress in LPS-induced septic cardiomyopathy through improving the function of antioxidant defenders such as GXP, SOD, and GPX 44. The antioxidant properties of irisin contributed also to decreased mitochondrial damage, as evidenced by normalized mitochondrial membrane potential and reduced mitochondrial ROS generation 44. Neferine, an alkaloid isolated from the green seed embryos of the lotus plant, was shown to mediate cardioprotection in LPS-treated mice through antioxidant and antiapoptotic effects mediated by induction of the PI3K/AKT pathway 45. The above research thus highlighted the functional importance of mitochondrial protection and antioxidant strategies in reducing sepsis-related myocardial injury. Based on this evidence, and the present experimental results, we propose that mitochondrial dysfunction and oxidative injury may function as fundamental intracellular transduction signals in response to sepsis-related cardiac inflammation.

The role of STK3 in the inflammatory response has been discussed in the setting of septic arthritis, chronic kidney disease, and sepsis-induced phagocyte activation. In a mouse model of *S. aureus*-induced septic arthritis, treatment with the synthetic retinoid derivative adapalene inhibits inflammation and promotes anti-inflammatory M2 polarization in macrophages by repressing AURKA-mediated WNT signaling and promoting Hippo signaling through activatory phosphorylation of STK3, STK4, and LATS1 46. Another study showed that mice with tubule-specific STK4/3 double knockout developed progressive fibrosis, inflammation, and tubular and glomerular damage, resulting in chronic kidney disease 47. Depletion of STK4/3 in myeloid cells was shown to increase susceptibility to bacterial sepsis and severe inflammation in mice. These effects were attributed to STK3-mediated mitochondrion-phagosome association, activation of Rac, and subsequent Toll-like receptor (TLR)-induced assembly of the TRAF6-ECSIT complex, leading to ROS production by phagocytes 19. In response to vascular injury, repression of STK3 by miR-155 was shown to trigger inflammation, oxidative stress, and vascular remodeling by promoting ERK1/2 phosphorylation 48. These findings indicate that STK3 are key mediators of inflammatory and bactericidal responses in different disease settings and cell types. In our study, LPS-induced septic cardiomyopathy enhanced the expression of STK3 in the mouse heart, while STK3/4 ablation *in vivo* or STK3 knockdown *in vitro* prevented cardiac damage and dysfunction by suppressing oxidative stress and preserving mitochondrial integrity. This finding highlights a novel role for STK3 in the pathological inflammatory response to sepsis and suggests that serum or cardiac STK3 levels might represent early markers to predict the extent of sepsis-related myocardial injury.

The Nrf2-dependent antioxidant response is a well-known protective mechanism against cardiovascular diseases and insults, including myocardial infarction 49, aortic stenosis 50, streptozotocin-induced cardiac toxicity 51, myocardial ischemia reperfusion injury 52, diabetic cardiomyopathy 53, and atherosclerosis 54. Likewise, several studies reported that during septic cardiomyopathy, Nrf2 activity protects heart function via multiple mechanisms 55-57. Therefore, augmentation of Nrf2-dependent antioxidant capacity is a promising strategy to sustain cardiac performance in cardiovascular disease. As an important mediator of Nrf2 degradation, KEAP1 has been found to promote the sepsis-involved cellular as well as organ damage. In LPS-treated adipose-derived stem cells, the expression of KEAP1 is significantly increased and correlates with a drop in the expression of Nrf2 as well as decreased levels of antioxidant enzymes 58. In septic cardiomyopathy, administration of naringin was shown to suppress KEAP1 expression and therefore restore the expression of Nrf2, leading to improved mitochondrial function 59. Our results showed that sepsis does not alter cardiac KEAP1 expression, but induces instead its phosphorylation at T85 through upregulation of STK3. Accordingly, transfection of a phosphodefective KEAP1 mutant restored Nrf2 expression, normalized mitochondrial function, and inhibited apoptosis in LPS-exposed cardiomyocytes. These findings hence suggest that KEAP1 phosphorylation may be a novel mechanism mediating blockade of the Nrf2-related antioxidant response during septic cardiomyopathy. Further studies assessing the impact of the expression of KEAP1 phosphorylation mutant proteins *in vivo* will help validate the functional importance of KEAP1 phosphorylation in septic cardiomyopathy.

In summary, our study provides *in vitro* and *in vivo* evidence that STK3-dependent KEAP1 phosphorylation impairs mitochondrial function and cardiomyocyte viability during septic cardiomyopathy by promoting Nrf2 degradation and hence decreasing Nrf2-dependent activation of an antioxidant response. These findings suggest that therapies aimed at preventing STK3 upregulation and KEAP1 phosphorylation may be provide effective cardioprotection in patients with septic cardiomyopathy.
